# Accumulating daily‐varied dose distributions of prostate radiation therapy with soft‐tissue–based kV CT guidance

**DOI:** 10.1120/jacmp.v13i3.3859

**Published:** 2012-05-10

**Authors:** Andrew Godley, Ergun Ahunbay, Cheng Peng, X. Allen Li

**Affiliations:** ^1^ Department of Radiation Oncology Medical College of Wisconsin Milwaukee WI 53226 USA

**Keywords:** deformable image registration, prostate radiation therapy, soft‐tissue–based registration, cumulative dose

## Abstract

Even with daily image guidance based on soft tissue registration, deviations of fractional doses can be quite large due to changes in patient anatomy. It is of interest to ascertain the cumulative effect of these deviations on the total delivered dose. Daily kV CT data acquired using an in‐room CT for five prostate cancer patients were analyzed. Each daily CT was deformably registered to the planning CT using an in‐house tool. The resulting deformation field was used to map the delivered daily dose onto the planning CT, then summed to obtain the cumulative (total delivered) dose to the patient. The delivered cumulative values of prostate $D_{100}$ on average were only 2.9% less than their planned values, while the PTV $D_{95}$ were 3.6% less. The delivered rectum and bladder V70s can be twice what was planned. The less than 3% difference between delivered and planned prostate coverage indicates that the PTV margin of 5 mm was sufficient with the soft‐tissue–based kV CT guidance for the cases studied.

PACS number: 87.55.km

## I. INTRODUCTION

Highly conformal radiation doses can be generated using intensity‐modulated radiation therapy (IMRT). However, the patient setup position and anatomy change daily, particularly in the prostate region, due to rectum and bladder filling. This interfractional change makes it challenging to deliver IMRT plans as accurately as required. Image‐guided radiation therapy (IGRT) repositions the patient to best match their planning position and internal anatomy, thereby reducing the interfractional variations. IGRT images with high soft tissue contrast, such as those from in‐room CT, permit soft tissue alignment. This offers an improvement over bony alignment based on 2D or 3D images with low soft tissue contrast. Further, the daily CT from an in‐room CT scanner has the diagnostic image quality used in treatment planning, allowing the recalculation of the dose delivered that day to elucidate the dose deviation from the plan caused by the interfractional changes. Daily dose deviations from the plan are seen to be substantial,^(^ ^1^ ^)^ despite soft‐tissue–based IGRT. This is due to target size and shape changes that cannot be alleviated by rigid shifts alone. It is desired to ascertain the cumulative effect of these daily dose deviations on the total delivered dose, to determine whether the treatment margins used are suitable to irradiate the tumor and avoid the critical structures.

The interfractional anatomic changes, such as those observed in prostate radiotherapy, require the cumulative dose over a fractionated delivery be calculated via deformable image registration. By deformably registering the daily IGRT CT to the original planning CT, a deformation field is determined that maps the voxels of the daily CT to the planning CT. This field can then deform the calculated daily dose distribution to the planning CT and is formatted as an array where each element represents the dose received by each planning CT voxel. Each deformed dose array can then be added to give the cumulative delivered dose and subsequently compared to the planned dose distribution.

Dose accumulation has been used to compare the efficacy of different treatment methods and to accurately investigate dose responses since the framework was defined.^(^ ^2^ ^)^ For example, it has been used to compare various IGRT techniques.^(^ ^3^ ^)^ Other studies have compared IGRT to adaptive planning^(^ ^4^ ^)^ and different alignment methods for IGRT and adaptive planning.^(^ ^5^ ^)^ Dose accumulation has also been used to determine tumor margins at which adaptive planning becomes beneficial over IGRT.^(^ ^6^ ^)^ These dose accumulation studies, however, used CT data acquired outside of the treatment room in a different session from treatment delivery. Therefore, the image acquired is not truly of the treated anatomy due to time elapsed and the patient moving from CT to linac. In addition, these studies sampled 10 to 15 out of 40 treatments.

More recent dose accumulation studies took advantage of in room imaging such as MV and kV cone beam or tomotherapy. However, the decrease in image quality affects the ability to address the interfractional variations and the electron density accuracy for dose accumulation. A study of prostate and head and neck radiotherapy tested MV cone‐beam–based dose accumulation.^(^ ^7^ ^)^ It has been reported that, even for kV cone‐beam CT, the Hounsfield Unit is different from that for a fan beam CT,^(^ ^8^ ^)^ limiting dose accumulation accuracy for this modality.^(^ ^8^ ^,^ ^9^ ^)^ Tomotherapy includes its own planned adaptive software to accumulate dose based on its MVCT that has been shown to be accurate.^(^ ^10^ ^)^ and was used to test the need for prostate adaptive radiotherapy.^(^ ^8^ ^)^ Lee et al.^(^ ^11^ ^)^ used all fractions to study accumulated tomotherapy doses for head and neck patients. The use of kV CT (e.g., from CT‐on‐rails) with the highest CT‐based soft tissue contrast can not only improve the patient alignment for interfractional variations, but can also lead to accurate dose accumulation.

There are, then, three avoidable areas of uncertainty in the dose accumulation: external room imaging, reduced image quality, and sampling. We desired to avoid these shortcomings. This work is based on available kV CT data acquired before each fraction using a CT‐on‐rails (CTVision, Siemens, Malvern, PA) immediately before treatment delivery during our routine soft‐tissue–based IGRT practice.

## II. MATERIALS AND METHODS

Daily CT data for five randomly selected prostate cancer patients treated with image‐guided, step and shoot IMRT were selected for this study. The original planning CT was obtained using a scanner (LightSpeed, GE Healthcare, Waukesha, WI). Immediately before each treatment, the daily CT was acquired using a CT‐on‐rails (Emotion 6/CTVision, Siemens). The patients were asked to have a full bladder and empty rectum prior to being treated, the same conditions under which they were simulated and planned. This was difficult to achieve in practice, as confirmed by the daily CT. The patient was repositioned based on a rigid image registration of the planning and daily CTs with soft tissue (the prostate rectum border) alignment. The treatment machine was a Siemens Primus linear accelerator. For this study, all available daily CTs were used. (Twenty‐four of the 210 daily CTs were not archived correctly and could not be included.)

The original treatment plans were produced from treatment planning systems (XiO, Elekta Ltd., CMS, Maryland Heights, MO or Panther, Prowess Inc., Concord, CA) with a prescription dose of 75.6 Gy in 1.8 Gy fractions to cover at least 95% of the prostate planning treatment volume (PTV). PTV margins of 5 mm around the prostate were used. The plans had seven or eight beams, with an average total of 36 segments. The delivery of the plans was tested before the first treatment using a MapCHECK diode array (Sun Nuclear, Melbourne, FL). Using a 3% dose difference or 3 mm distance to agreement, the measured gamma index^(^ ^12^ ^)^ was greater than 99% for all plans.

To calculate the delivered dose of each fraction, the pretreatment CT of the fraction was loaded into the respective planning system. The original treatment beams were applied to the daily CT, incorporating the daily shifts determined by the therapists, and the original treatment parameters (e.g., monitor units). An electron density calibration curve was measured specifically with our CT‐on‐rails.

Each daily CT was deformably registered to the planning CT using a newly developed deformable registration tool. This tool was developed in‐house with the Insight Toolkit,^(^ ^13^ ^)^ using a fast symmetric demons algorithm.^(^ ^14^ ^)^ Due to the large differences seen between the daily CTs driven by changes in bladder and rectum filling, the bladder and rectum must be masked in all images to guide the intensity‐based demons registration. This also removes the difficulty the demons algorithm has with rectal gas, which may be present in one image but not the other. The voxels of the rectum and bladder were replaced with a uniform intensity of 1000 and −1000 HU, respectively, in both the daily and planning CT. The masks were created from physician contours and the same physician contoured all scans. Work is ongoing to determine the rectum and bladder masks with image segmentation. Further details of the deformation and masking are available.^(^ ^15^ ^)^

The average Dice's coefficient or DC^(^ ^16^ ^)^ between the rectum, bladder, and prostate contours of the daily and planning CT are given for the five patients in Table 1. The “Initial” column refers to the agreement after rigid registration of the bony anatomy, elucidating the large daily soft tissue variation observed. “Final” is after the deformable registration. The greater than 93% DC for rectum and bladder and 84% for prostate imply acceptable registration of the daily CTs to the plan, as this is consistent with their interobserver contour agreement. (See Table 3 in Mazonakis et al.^(^ ^17^ ^)^) This successful registration is evidenced despite initial organ DC below 60%. The agreement was confirmed by visual comparison of each deformed daily CT with the planning CT.

**Table 1:** Average Dice's^(^ ^16^ ^)^ coefficient % (mean ± standard deviation) between daily and planning CT organ contours after bony anatomy alignment (Initial) and deformable registration (Final).

	*Rectum*		*Bladder*		*Prostate*	
*Patient*	*Initial*	*Final*	*Initial*	*Final*	*Initial*	*Final*
1	59.4±4.1	94.2±0.5	91.1±2.1	96.7±0.8	85.2±4.8	88.6±3.4
2	70.5±3.4	93.5±0.9	83.0±6.9	98.2±0.2	80.4±2.9	84.0±2.9
3	71.9±5.6	96.1±0.4	85.4±3.3	95.2±0.7	79.7±6.5	89.4±1.3
4	69.3±5.8	96.1±0.3	83.4±3.6	98.8±0.1	70.4±0.9	88.6±1.9
5	65.8±8.9	96.4±0.3	87.3±3.5	96.5±0.5	79.2±6.4	85.7±2.9

The deformation field resulting from the registration was then used to map the daily calculated dose to the planning CT. This gives the dose delivered to each voxel of the planning CT, from each treatment fraction. These dose arrays were then simply summed to obtain the cumulative (total delivered) dose to the patient. The planned, delivered daily, and cumulative dose distributions and dose‐volume histograms (DVHs) were compared.

The following metrics were used to evaluate the differences between the planned and delivered doses. The prostate $D_{100}$ (dose in Gy delivered to 100% of the target volume) was used to assure target coverage was maintained, while the PTV $D_{95}$ was used as it is the prescription point. The $V_{70}$ (percentage of organ volume receiving 70 Gy) for the rectum and bladder were included as $V_{70}$ has been associated with rectal toxicity^(^ ^18^ ^)^ and a constraining value for the bladder.^(^ ^19^ ^)^ Rectal and bladder mean dose were also compared.

To test for errors in the dose deformation implementation, the plan dose was mapped from the planning CT to the planning CT (i.e., the planning CT was registered to itself, and the plan dose deformed by the resulting (identity) deformation field). This is to test for errors or excessive rounding within the dose deformation process. The plan dose is therefore the expected output, and indeed, there were no discernable differences between the mapped dose and the original. Additionally, a delivered dose was mapped from its daily CT to the planning CT and then back to the daily CT. The only differences seen were in the penumbra of the beams, the high dose gradient region. These differences were less than 5% and caused less than 0.2% difference in the dose volume parameters considered.

## III. RESULTS

**Figure 1:**
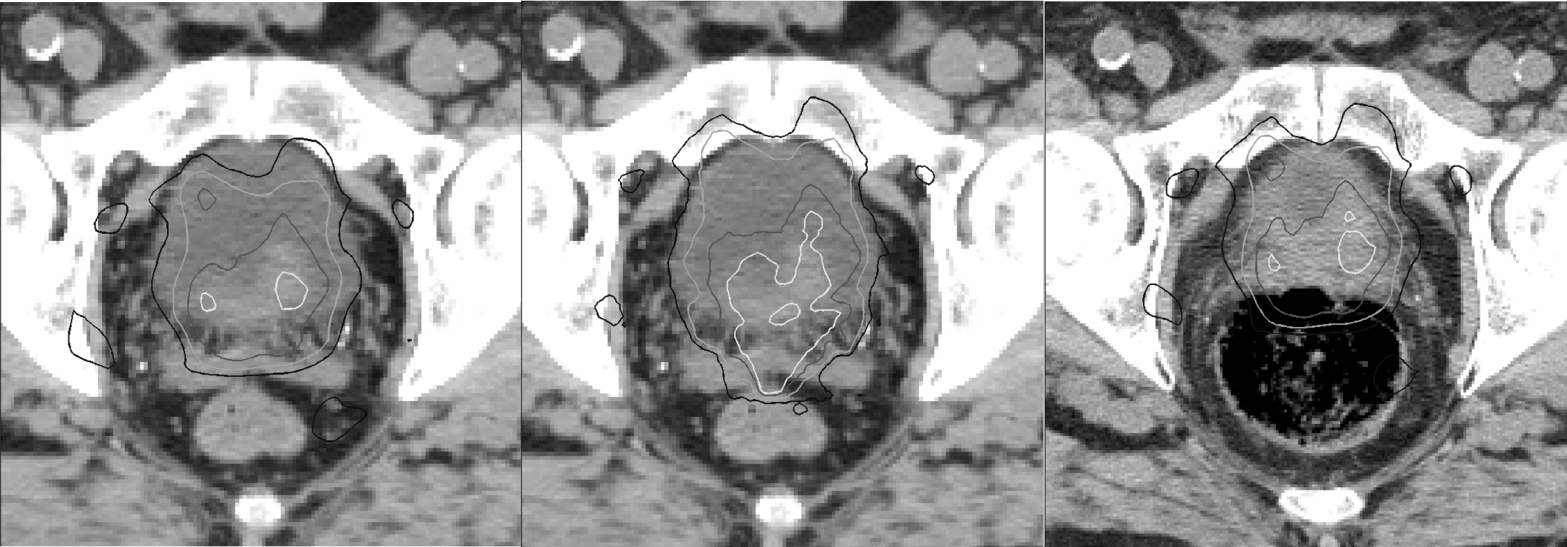
An example of dose deformation for a fraction with a large organ deformation, showing the planned (left) and deformed (middle) doses overlaid on the plan CT and the reconstructed dose (right) overlaid on the daily CT. Isodose lines are 105% (white), 100% (dark gray), 90% (light gray), and 80% (black) of the prescription dose.

An example of dose deformation is illustrated in Fig. 1. The delivered dose distribution for a fraction with a large organ deformation was compared to the planned dose distribution. Both dose distributions were overlaid on the planning CT. The reconstructed dose on the daily CT was also given. The large change in the rectal filling caused the rectum and the seminal vesicles to enter the high‐dose region, as seen in the deformed dose distribution.

**Figure 2:**
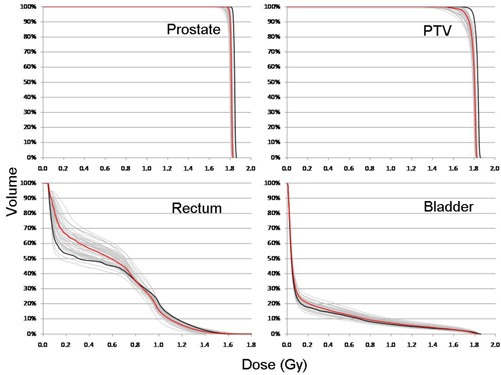
DVHs for each treatment fraction (gray) overlaid with the planned DVH (thick black), and cumulative scaled to one fraction (red) for the prostate, PTV, rectum, and bladder for patient 5 who had large organ variation.

Figure 2 shows DVHs for each treatment fraction overlaid with the planned DVH of the prostate, PTV, rectum, and bladder for a patient with large organ variation, patient 5. Also shown is the cumulative dose, scaled down to one fraction. All the DVHs were calculated using the plan contours after the fractional doses were deformed to the planning CT, (i.e., differences due to changes in organ shape only, not contouring differences). The DVHs highlight the range of doses delivered each day to the various organs over the course of treatment. A considerable spread is seen in the DVHs, particularly for the PTV, rectum, and bladder. The cumulative DVHs are consistent with these daily doses.

**Figure 3:**
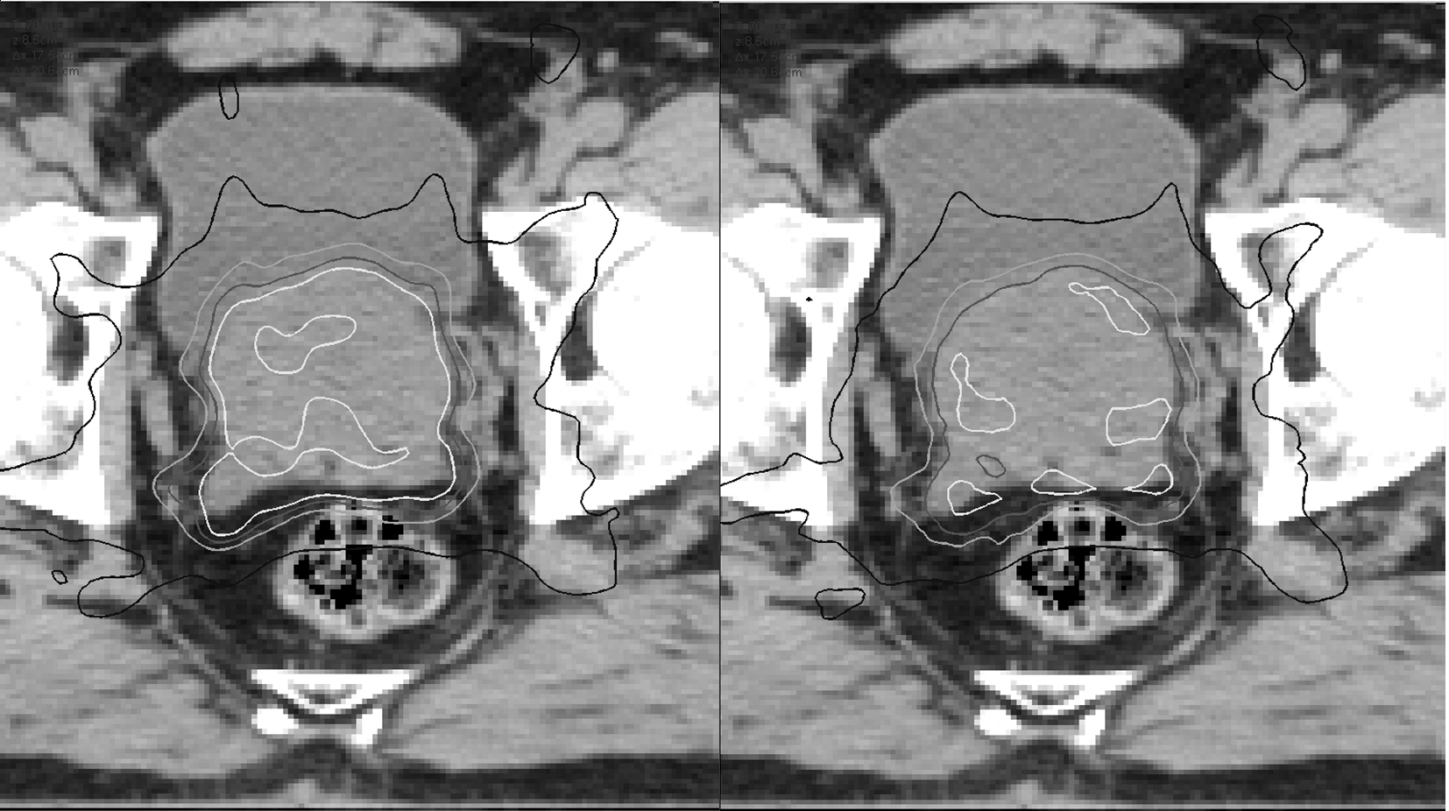
Planned vs. cumulative delivered dose for a patient with large interfraction variation. The planned (left) and cumulative (right) dose distributions are overlaid onto the planning CT. Isodose lines are 105% (white), 100% (dark gray), 90% (light gray), and 67% (black) of the prescription dose.

An example delivered cumulative dose distribution calculated with deformable registration was compared to the planned dose distribution in Fig. 3. Both dose distributions were overlaid on the planning CT. Deviations between the planned and delivered doses were seen. Also noticed was the reduction of the 105% isodose line.

**Figure 4:**
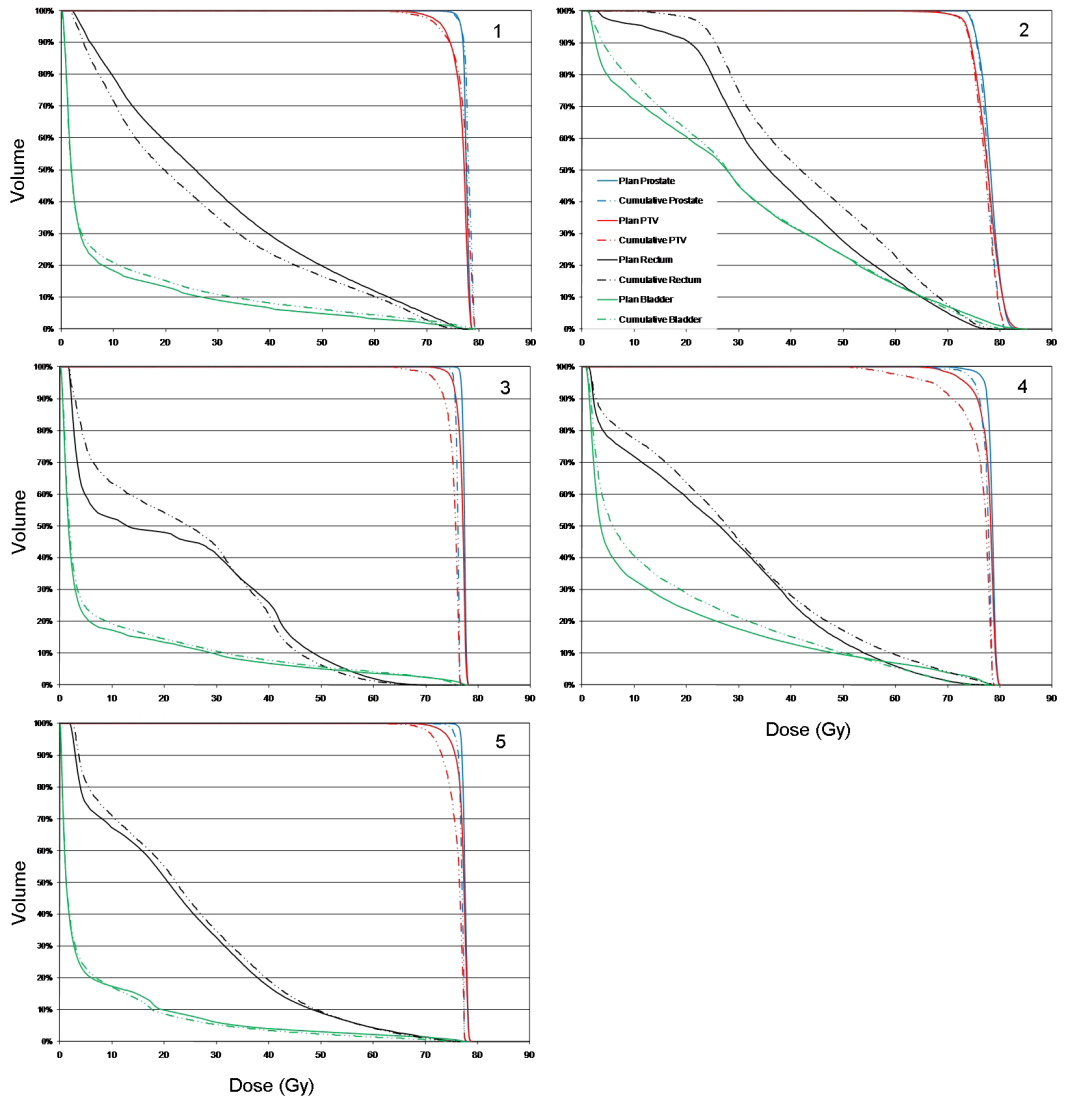
DVH comparison of planned and cumulative delivered dose for the five patients.

The cumulative DVHs were compared with the plan DVHs in Fig. 4 for all five patients studied. The DVHs for prostate are similar, indicating that the PTV margin used was reasonable. However, a shoulder appears on the PTV DVHs, due to the underdosing at the edges of the PTV. The delivered DVHs for the more anatomically variable rectum and bladder reveal a higher change from their corresponding planned DVHs.

Table 2 compares the cumulative delivered doses at the completion of treatment to the planned doses for all five patients in terms of the $D_{100}$ and $D_{95}$ for the prostate and PTV, and the mean dose and $V_{70}$ of the rectum and bladder. Negative values indicate an underdose with respect to the plan.

**Table 2:** Planned, delivered, and difference for the dose‐volume quantities of the targets and organs at risk.

		*Patient*	*1*	*2*	*3*	*4*	*5*
Prostate	D_95_	Plan	76.5	75.0	76.7	77.1	76.9
	(Gy)	Delivered	76.8	75.0	75.3	75.1	75.9
		% Diff	0.5	0.0	−1.8	−2.6	‐1.3
	D_100_	Plan	71.8	74.0	75.2	69.1	73.7
	(Gy)	Delivered	72.8	73.2	73.4	66.0	68.0
		% Diff	1.4	1.1	−2.4	−4.5	−7.7
	DVH Variation		0.6	0.6	1.0	1.2	0.9
PTV	D_95_	Plan	73.0	73.6	75.2	73.4	74.6
	(Gy)	Delivered	72.4	73.4	71.8	67.1	71.8
		% Diff	−0.7	−0.3	−4.5	−8.6	−3.8
	D_100_	Plan	60.8	57.6	68.7	63.0	65.9
	(Gy)	Delivered	59.6	64.2	62.9	50.4	58.2
		% Diff	−2.0	12.3	−8.4	−20.2	−11.7
	DVH Variation		0.7	0.6	1.7	2.4	1.6
Bladder	Mean	Plan	8.4	30.2	8.2	14.4	6.6
	(Gy)	Delivered	9.8	30.9	9.0	16.3	6.2
		%Diff	16.7	2.0	9.6	13.0	−7.2
	V_70_	Plan	1.7	7.1	2.4	3.9	1.4
	(%)	Delivered	2.6	6.2	2.4	1.1	0.6
	(%)	Abs. Diff	0.9	−0.9	0.0	−2.8	−0.8
	DVH Variation		1.5	1.5	1.1	3.5	0.9
Rectum	Mean	Plan	29.7	39.1	21.8	26.7	23.1
	(Gy)	Delivered	26.4	43.5	23.6	29.4	24.3
		%Diff	−11.8	11.1	8.5	9.8	5.5
	V_70_	Plan	4.7	5.1	0.0	1.3	1.4
	(%)	Delivered	3.0	7.7	0.0	3.8	1.1
	(%)	Abs. Diff	−0.3	2.6	0.0	2.5	−1.3
	DVH Variation		4.8	6.6	4.0	3.3	1.7

It is shown by Table 2 and Fig. 4 that for the five patients studied, the delivered prostate $D_{100}$ is under the planned value by an average of 2.9%. For PTV $D_{95}$, the delivered values were on average 3.6% less than the plan. These small deviations for the prostate $D_{100}$ imply that the PTV margin used was adequate. There is little expected clinical significance for such dose deviations. The changes in PTV coverage were mainly due to the daily shape variation, pushing different edges of the PTV outside the prescription dose region, reducing its overall coverage.

The delivered $V_{70}$ values of the bladder and rectum can be decreased or increased as compared to the planned values. Decreases in $V_{70}$ occur, particularly for the bladder, due to more advantageous anatomy delivered to than used in planning (e.g., less bladder pressing against the prostate). However, all delivered $V_{70}$s were less than the QUANTEC recommended values of 20% and 35% of the rectum and bladder volumes, respectively,^(^ ^18^ ^,^ ^19^ ^)^ and less than the 15% of organ volume used at our institute. Thus the changes seen are unlikely to be clinically significant. The change in $V_{70}$ is dramatic due to small volumes of bladder and rectum in a high dose gradient region. Mean dose was, therefore, included to provide a more robust indication of the change in organ dose. These saw a smaller variation from the planned values of −11% to +17%.

**Figure 5:**
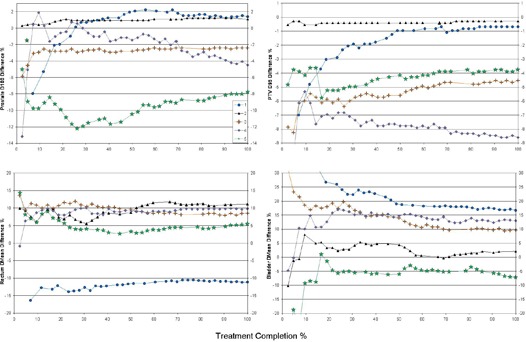
Evolution of delivered dose over the course of treatment. A comparison of the percentage difference between the delivered dose accumulated up to the given fraction and the total planned dose for the five patient cases.

Further insight from the deformed dose data can be gleaned by observing the delivered dose as a function of fraction completed (Fig. 5). The cumulative dose was determined as each fraction is added (e.g., a $D_{95}$ in the figure at 20% treatment completion represents the dose delivered over the first 20% of the fractions). The cumulative dose is scaled by the percentage of fractions included so far, so as to always be directly comparable to the planned dose of the entire treatment course. The same percentage difference of delivered and planned dose quantities is used as in Table 2 (i.e., the values at 100% treatment completion comprise Table 2). These plots represent the evolution of the delivered dose as the treatment progresses.

Initially, the disagreement with the plan can be large, depending on the anatomy of the patient on the first days. The difference in delivered dose begins to decrease and stabilize as more fractions are accumulated, as areas of organs that were in a lower dose region move to a higher dose region and vice versa. The rectum and bladder show a larger variation that lasts further into treatment but eventually settles to a consistent value. This dose variation is again due to the larger change in bladder and rectum size compared to the prostate and their location at the periphery of, rather than within, the high dose region.

## IV. DISCUSSION

The accumulated delivered doses were calculated for five patients via deformable registration of the daily kV CTs to the planning CT. This study is different from studies reported previously in the following aspects: i) the use of in‐room kV CTs with the same image quality as the planning CT eliminates the uncertainties associated with using cone‐beam CT for structure delineation and electron density for dose calculation, and ii) the use of daily CTs, accurately capturing the entire interfractional variations. The present study provides the cumulative delivered doses that were found to be different from the planned doses.

Comparing delivered doses using individual treatment CTs can introduce errors due to the intraobserver contouring variation, which can be 5%–7% in the prostate region.^(^ ^17^ ^)^ Changes in delivered doses can be overestimated by such variations in organ delineations. It is more accurate to compare the deformed doses with one set of contours, the plan contours, eliminating the need for contouring on daily CTs. For this reason, the current study was carried out by deforming all daily doses to the planning CT.

For the patients studied, the less than 3% average difference between delivered and planned prostate coverage ($D_{100}$) indicates that the 5 mm PTV margin was sufficient for the soft‐tissue–based daily repositioning using the kV CT (e.g., CT‐on‐rails). The delivered doses (mean and $V_{70}$) for rectum and bladder, vary more substantially from patient to patient, but are still within treatment limits. These variations are reflected in the DVHs of Fig. 4. As a quantitative estimate of this, the average variation between the planned and delivered DVH is included in Table 2. The prostate DVH varies by 0.6% to 1.2%, the bladder by 0.9% to 3.5% and the rectum from 1.7% to 6.6%. This is to be expected as Table 1 shows that the highest disagreement between the treated and planned volumes is for the rectum. Future work will investigate treatment with an empty bladder, which should be more reproducible and comfortable. Additionally, it would be desirable to simulate and treat with an empty rectum, which may require a change of diet to reduce rectal gas.

The dose differences determined herein may not necessarily be applied to treatments using different IGRT imaging modalities such as cone‐beam CT and MV CT where the image quality may not provided sufficient alignment accuracy (e.g., alignment may have to be based on skeleton and not soft tissue). Previous studies using cone‐beam CT and MV CT reported higher deviations from planned doses.^(^ ^4^ ^,^ ^8^ ^,^ ^9^ ^)^

A source of error to the dose accumulation not accounted for is the intrafraction motion. The daily CT is taken 2–3 minutes before the treatment, and the fully sequenced IMRT can take up to 7–8 minutes to deliver. In this 10 minute period, the patient anatomy can be different from what was imaged, and hence, from what was used to calculate the delivered dose. According to cine MR measurements, the mean prostate motion is zero with a standard deviation of 1 mm over a 9 minute scan.^(^ ^20^ ^)^ Measurements with electromagnetic transponders reveal that the chance for prostate displacement > 3 mm is less than 14% during ~ 10 minute treatments.^(^ ^21^ ^)^ This small intrafraction motion, which is well within the PTV margin, implies the dose determined using the pretreatment CT is practically acceptable, given the current lack of real‐time imaging for dose reconstruction. Cine MRI for the bladder shows greater changes in shape over the course of 14 minutes.^(^ ^22^ ^)^ However, this change is far away from the high dose region, and thus should have a small affect on the bladder dose calculation.

The CT‐on‐rails provides additional radiation that is not accounted for in the treatment planning or the dose accumulation. The imaging dose must be managed,^(^ ^23^ ^)^ and is minimal here at a level below that of a regular kV CT due to the fact that only about 40 slices are imaged.

Schulze et al.^(^ ^5^ ^)^ investigated dose delivered to nine prostate patients, but only used 10 CT sets acquired outside the treatment room. They tested a number of alignments, margins, and replanning, and observed 0% to 1.5% underdose in prostate, and −9% to 16% and 8% to 5% changes, respectively, in equivalent uniform dose for the bladder and rectum, as compared to the plan. These findings are generally consistent with our data for prostate coverage and rectum and bladder mean doses.

Peng et al.^(^ ^1^ ^)^ looked at the daily dose variation in prostate radiotherapy without studying the cumulative doses. They observed a daily reduction of prostate $D_{100}$ of no more than 3% for 94.1% of fractions, in line with our cumulative $D_{100}$ value of 2.9%. The rectum and bladder $V_{70}$s typically increased by up to 26% daily, while the $V_{45}$ swung between ±30%. Our cumulative rectum and bladder doses show a slight upward trend (5%–7%) in the mean dose (14 Gy originally planned to the bladder, 28 Gy to the rectum), and more erratic changes than the $V_{70}$s in the Peng study. Both studies show that changes from the planned doses to the delivered doses are larger for the rectum and bladder than those for the target.

Adaptive radiation therapy, such as online replanning,^(^ ^24^ ^)^ has the capability to fully address the interfractional variations, reducing or eliminating PTV margins. Cumulative dose compiled during treatment, akin to Fig. 5, would be useful in ascertaining when adaptive treatment needs to occur. For example, in order to replan for a 5% reduction in $D_{100}$ of the prostate or a projected $V_{70}$ greater than institutional limits.

## V. CONCLUSIONS

Interfractional variations during prostate radiotherapy can result in substantial difference between the delivered and planned doses, particularly in critical structures. The less than 3% difference between delivered and planned prostate coverage indicates that the PTV margin of 5 mm was sufficient with the soft‐tissue–based kV CT guidance for the cases studied. Further reduction in PTV margin would require adaptive approaches, such as online replanning, to address the large interfractional anatomic variations.

## ACKNOWLEDGMENTS

This work is supported partially by MCW Cancer Center Fotsch Foundation.
